# Profile of a new extended range-of-vision IOL: a laboratory study

**DOI:** 10.1007/s00417-021-05426-3

**Published:** 2021-10-04

**Authors:** Daniele Tognetto, Rosa Giglio, Chiara De Giacinto, Marco R. Pastore, Gabriella Cirigliano, David P. Piñero, Gianluca Turco

**Affiliations:** 1 Department of Medicine, Surgery and Health Sciences, University of Trieste, Trieste, Italy; 2 Department of Optics, Pharmacology and Anatomy, University of Alicante, Alicante, Spain

**Keywords:** Cataract, Presbyopia, Intraocular lens, Monofocal, Extended depth of focus, Extended range of vision

## Abstract

**Purpose:**

To evaluate the surface profile of a new-generation extended range-of-vision intraocular lens (IOL) and to compare it with that obtained for a monofocal IOL based on the same platform.

**Methods:**

Prospective, experimental, laboratory study comparing the surface profile of the DFT015 (AcrySof IQ Vivity; Alcon Laboratories, Inc.), a new-generation presbyopia-correcting IOL, with the profile of the SN60WF (AcrySof IQ; Alcon Laboratories, Inc.), an aspheric monofocal IOL based on the same platform. Raw profiles were obtained using contact profilometry. The best-fit form was then subtracted from each raw profile to highlight potential differences.

**Results:**

No significant differences were appreciated in raw profiles. On the contrary, after form removal, the new extended range-of-vision IOL showed a peculiar profile characterized by the presence of two altitudinal symmetrical changes in the order of 1 µm, localized in the central portion of the optic.

**Conclusions:**

The new-generation extended range-of-vision IOL evaluated showed a smooth change of its surface compared to the same platform monofocal IOL. The altitudinal changes blended in the central design of the new presbyopia-correcting IOL, although micrometric, might play a crucial role in creating a continuous focal range while minimizing visual disturbances.


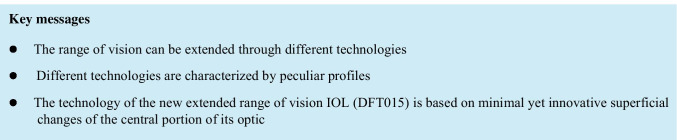


## Introduction

Cataract surgery has evolved from a rehabilitative procedure to a refractive procedure. The increased demand for higher and higher spectacle independence after the surgery has led to the development of innovative technologies, in particular in the field of intraocular lenses (IOLs). As reported by a recent statement of the European Society of Cataract & Refractive Surgeons on functional vision, the achievement of a certain distance visual acuity threshold is not always related to the patients’ self-assessed vision improvement [1]. Although effective in restoring excellent distance vision, monofocal IOLs might not always meet the expectations of cataract patients for their post-operative refractive outcome. Multifocal IOLs (MFIOLs) were designed to improve spectacle independence at different distances, but they have been associated with some concerns regarding contrast sensitivity reduction and increased photic phenomena [2–5]. Thus, extended depth of focus (EDOF) IOLs have been added to the range of the different solutions available for pseudophakic presbyopia correction. These IOLs have been designed to deliver an extended range of vision without increasing the risk of unwanted side effects such as photic phenomena [6]. In this study, we analysed the anterior surface profile of one of the latest presbyopia-correcting IOLs (PCIOLs) based on an innovative non-diffractive technology.

## Methods

### Intraocular lenses

The following IOLs were included in the analysis: DFT015 (AcrySof IQ Vivity, Alcon Laboratories, Inc.) and SN60WF (AcrySof IQ; Alcon Laboratories, Inc.). The DFT015 is an extended range-of-vision IOL based on a non-diffractive technology called X-wave (X-wave EDOF) [Alcon Data on File, 2019–2020; AcrySof® IQ Vivity™ Extended Vision IOL Directions for Use. Alcon Laboratories, Inc.; 2020]. Specifically, it uses a central 2.2-mm optical zone, containing two non-diffractive transition elements changing the wavefront of the central light beams to elongate the depth of focus. The anterior surface of the IOL is also designed with negative spherical aberration to compensate for the positive spherical aberration of the cornea. The result is a continuous extended focal range with monofocal visual disturbances profile [Alcon Data on File, 2019–2020; AcrySof® IQ Vivity™ Extended Vision IOL Directions for Use. Alcon Laboratories, Inc.; 2020]. The SN60WF is an aspheric biconvex monofocal IOL, with a reported 0.20 μm of negative spherical aberration. The DFT015 and the SN60WF share the same ultraviolet and blue light filtering acrylate/methacrylate copolymer. Their optic diameter is 6 mm, and the overall length is 13 mm. Both IOLs have modified L-loop haptics, with no angulation and a square edge interrupted at the optic-haptic junction. Their refraction index is 1.55 at 35°. The IOL power range varies from + 15.0 to + 25.0 D (step 0.5 D) for the DFT015 and from + 6.0 to + 30.0 (step 0.5 D) for the SN60WF. The same power was used for all IOLs included in the analysis (+ 20D). For each model, three IOLs were consecutively tested.

### Measurement method

The surface profile was obtained using a profilometer (Talysurf CLI 1000, Taylor Hobson) operating in contact mode, with an inductive type of contact stylus sensor. The stylus vertical movement on the specimens’ surfaces is converted into an electrical signal by an inductive gauge. This profilometer has shown a high resolution and a high accuracy [7, 8]. For the analysis of the raw profiles, the IOLs were positioned on a micrometric moving platform, driven at a pre-defined scanning speed and spatial resolution. The acquisition speed was 50 micron/s with a lateral resolution of 50 nm and a vertical resolution of 10 nm. The sampled three-dimensional digital coordinates of the investigated surface were stored and analysed using the TalyMap software. The scanned surface profiles were derived from the conversion of those coordinates into two-dimensional (2D) profile graphs (raw profiles). A nominal shape was subtracted by the measured profile, and the best-fit form was removed from each profile as previously described [8].

## Results

**Fig. 1:**
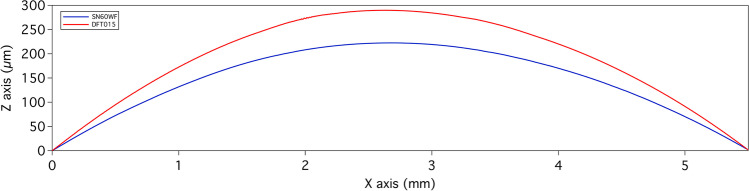
The 2D representations show the central surface waviness (raw profiles) obtained by inductive contact stylus measurements of the SN60WF (blue line) and of the DFT015 (red line)

Profiles extracted by inductive measurements are shown in Fig. 1. No significant differences could be appreciated in the 2D representation of the profiles of the monofocal IOL (SN60WF) and of the extended range-of-vision IOL (DFT015).

**Fig. 2:**
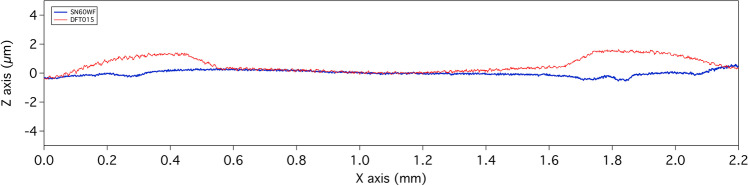
Subtraction lines obtained after the removal of the best-fit form from the raw profile of the SN60WF (blue line) and of the DFT015 (red line)

Nevertheless, after removal of the best-fit form from the raw profiles, as expected, the standard monofocal IOL showed an almost flat subtraction line, meaning that at least in the central 2.5 mm the profile was almost perfectly spherical and compatible with the central portion of an aspherical surface (Fig. 2). The extended range-of-vision IOL subtraction line showed a symmetrical deviation from the ideal central circular shape, indicating a smooth altitudinal change, in the range of 1 µm, located in the paracentral portion of the optic (Fig. 2).

## Discussion

In the current study, we have analysed the surface profile of a non-diffractive presbyopia-correcting IOL (DFT015), characterized by the introduction of two surface elements on its optic. Only a few data on DFT015 optical bench and clinical outcomes have been published so far due to its relatively recent introduction into clinical practice [9–13].

In a prospective case series involving 108 eyes (54 patients) implanted with the DFT015, the uncorrected intermediate visual acuity (VA) at 60 cm was 0.06 ± 0.08 LogMAR at the 3-month postoperative follow-up [11]. In the same study, it was reported an excellent refractive outcome for far but near vision required a spherical addition of at least + 1.0 D [11]. Furthermore, as with other EDOF, it has been suggested that a minor myopic target in the non-dominant eye (0.50–1.0 D) could increase the binocular defocus curve and hence the functional range of vision of the DFT015 substantially [10]. Schmid and Borkenstein investigated the higher order aberrations (Zernike polynomials up to the 10th order) of four new-generation IOLs, including DFT015, using a Shack–Hartmann sensor [13]. The wavefront pattern observed for the DFT015 was made of a pronounced negative spherical aberration (SA) 4–0 of − 1.01 λ, a SA 6–0 of 0.27 λ and a SA 10–0 of − 0.21 λ. For lower order aberrations, small astigmatism was recorded. The authors concluded that the magnitude of DFT015’s SA modification could significantly increase the depth of focus [13].

In the present study, the raw profile of the DFT015 IOL showed no significant differences in the central portion from the same platform monofocal IOL (SN60WF). After the form removal, the DFT015 IOL profile showed the presence of two symmetrical elements in the central part of the optic, suggesting a change of refraction in the paracentral area. Although the resolution of the profilometer is in the range of nanometres, it was not able to macroscopically detect the point-by-point fine changes on the IOL surface at the first analysis. These could be explained because differently from traditional diffractive technologies, these changes seem exceptionally smooth and blended on the overall surface. In a previous laboratory study, we have analysed the profile of different IOLs including two PCIOLs: the ZXR00 (TECNIS Symfony, Johnson and Johnson Vision) and the Mini Well (Sifi) [8]. The ZXR00 diffractive pattern, an example of a typical echelette design, could be easily detected by contact profilometry with the IOL showing a saw-toothed raw profile compared to the same platform monofocal IOL (ZCB00, TECNIS-1-piece, Johnson and Johnson Vision) [8]. As with the DFT015 and SN60WF raw profiles, the Mini Well raw profile, an EDOF based on spherical aberration, appeared to be indistinguishable in the central portion from the same platform monofocal IOL (Mini 4). However, after the best-fit form removal, the spherical aberration–based IOL showed a hill and valley morphology with a central steepening in the range of 10 µm [8].

The presence of the transition elements featured by the DFT015 might explain the significant improvement in the refractive outcomes of the DFT015 compared to the SN60WF, observed by other authors [9–11]. Further clinical trials will be necessary to confirm the refractive outcome by real-word experience and to compare this IOL with other PCIOLs.

The trend of abandoning multifocality in favour of improving intermediate vision has resulted in the development of new concept IOLs with gradual and subtle changes in the IOLs’ superficial geometry that may provide better clinical performance than classic diffractive designs, particularly in terms of unwanted photic phenomena. This hypothesis should be confirmed by future optical bench and clinical studies. The knowledge of the available options for pseudophakic presbyopia correction is crucial, together with patient selection, to avoid post-operative dissatisfaction [6]. Understanding the structural differences that characterize distinct technologies is useful to forecast and to critically analyse the clinical outcomes. Future studies seem necessary to better understand the connection between the surface profile of this IOL, its optical behaviour and consequently, the final clinical outcome provided.
